# Enteral Nutrition in Term Infants with Congenital Heart Disease: Knowledge Gaps and Future Directions to Improve Clinical Practice

**DOI:** 10.3390/nu13030932

**Published:** 2021-03-13

**Authors:** Silvia Martini, Isadora Beghetti, Mariarosaria Annunziata, Arianna Aceti, Silvia Galletti, Luca Ragni, Andrea Donti, Luigi Corvaglia

**Affiliations:** 1 Department of Medical and Surgical Sciences, University of Bologna, 40138 Bologna, Italy; silvia.martini9@unibo.it (S.M.); isadora.beghetti@studio.unibo.it (I.B.); mariarosa.annunziata@studio.unibo.it (M.A.); silvia.galletti4@unibo.it (S.G.); luigi.corvaglia@unibo.it (L.C.); 2 Neonatal Intensive Care Unit, IRCCS Azienda Ospedaliero-Universitaria di Bologna, 40138 Bologna, Italy; 3 Pediatric Cardiology Unit, IRCCS Azienda Ospedaliero-Universitaria di Bologna, 40138 Bologna, Italy; luca.ragni@aosp.bo.it (L.R.); andrea.donti@aosp.bo.it (A.D.)

**Keywords:** congenital heart disease, enteral nutrition, term infants, perioperative feeding management, human milk, necrotizing enterocolitis, prostaglandin, patent ductus arteriosus

## Abstract

Optimal nutrition is essential to improve short- and long-term outcomes in newborns with congenital heart disease (CHD). Nevertheless, several issues on nutritional management and concerns about the potential risk of complications related to enteral feeding exist. This narrative review aims to summarize and discuss the available literature on enteral feeding in term infants with CHD. A wide variability in feeding management exists worldwide. Emerging approaches to improve nutritional status and outcomes in infants with CHD include: implementation of a standardized enteral feeding protocol, both preoperative and postoperative, clearly defining time of initiation and advancement of enteral feeds, reasons to withhold, and definitions of feeding intolerance; early minimal enteral feeding; enteral feeding in stable term infants on hemodynamic support; evaluation of enteral feeding in term infants with umbilical arterial catheters and during prostaglandin infusion; assessment and support of oro-motor skills; and promotion and support of breastfeeding and provision of mother’s own milk or donor milk when mother’s own milk is not available. As evidence from term infants is scarce, available observations and recommendations partially rely on studies in preterm infants. Thus, well-designed studies assessing standardized clinically relevant outcomes are needed to provide robust evidence and shared recommendations and practices.

## 1. Introduction

Congenital heart disease (CHD) is one of the most common anomalies of human development, with an estimated incidence of 4 to 10 cases per 1000 live births and of about 6 per 1000 live births for moderate and severe forms [1,2,3]. Most infants born with CHD have a normal weight for gestational age at birth [4], but a relevant proportion of them (between 15% and 41%) [5,6] develop malnutrition and growth deficiencies during the first months of life, this leading to delayed or complicated cardiac surgery [5,7]. Several factors are known to contribute to the development of malnutrition in infants with CHD. The increase in sympathetic activity, work of breathing, and the presence of congestive cardiac failure overall contribute to increase the metabolic demand [7]. Failure to thrive is more frequent and severe in neonates with cyanotic CHD compared with non-cyanotic CHD, and it is further worsened by pulmonary hypertension [5]. Structural abnormalities of the gastrointestinal (GI) tract, which occur in 4.2% of children with CHD, gastroesophageal reflux, and food intolerance can also contribute to inadequate nutrient intake and consequent poor growth [8,9]. Moreover, associated genetic syndromes such as Down syndrome, Di George syndrome, Turner syndrome, and trisomy 13 and 18 are independently associated with abnormal growth [10]. A high risk of growth failure persists in the postoperative period [11]; while successful surgical treatment of CHD improves neonates’ long-term survival, catch-up growth is impaired in a relevant proportion of these infants [12].

The consequences of malnutrition during critical illness and perioperatively can be more severe in young children and neonates, since in this population greater caloric and nutrients intakes are required to promote an adequate growth and psychomotor development [13]. Hence, suboptimal nutrition in CHD infants has been associated with poorer clinical outcomes: energy and protein deficiency worsens stress-induced catabolic response, impairs wound healing, affects myocardial and muscle function, and may increase the rate of postoperative complications [10]. Moreover, growth failure in infants with CHD has been specifically associated with long-term cognitive impairment [14,15].

Although optimal nutrition is considered essential to improve short- and long-term outcomes in newborns with CHD, large well-designed randomized controlled trials assessing clinically relevant outcomes and interventions are scarce. Position statements and clinical recommendations summarizing the existing evidence have recently become available; nevertheless, several relevant questions on the optimal nutrition in infants with CHD remain unanswered. The aim of this narrative review is to summarize currently available literature on enteral feeding in term infants with CHD, to highlight open issues, and to identify future directions for the improvement of clinical practice.

## 2. Literature Review

Medline, via PubMed, (http://www.ncbi.nlm.nih.gov/pubmed/, accessed on 8 March 2021), the Cochrane Library (http://www.cochranelibrary.com/, accessed on 8 March 2021), and Embase (http://www.embase.com/, accessed on 8 March 2021) were interrogated for studies published before 30 November 2020. Search strings were built up by combining all the terms related to enteral feeding and congenital heart disease, using PubMed MeSH terms, free-text words, and their combinations to be as comprehensive as possible. Similar criteria were used for searching the Cochrane Library and Embase. The review was restricted to English-written studies. The reference lists of retrieved studies were searched for further relevant papers. Retrieved papers were categorized according to the following topics, relevant to enteral feeding management in term infants with CHD: preoperative feeding management; postoperative feeding management; impact of breastfeeding and type of milk; and feeding issues.

## 3. Preoperative Feeding Management

Preoperative feeding practices in infants with CHD are very heterogeneous across Intensive Care Units (ICUs) worldwide, and a shared feeding strategy defining the timing for feeding introduction, feeding modality, or optimal intakes is lacking [16,17]. In almost half of European Centers, enteral feeding in infants with CHD is avoided before surgery due to concerns about GI complications and splanchnic ischemia [18,19].

The variation in feeding practices and the lack of a consensus regarding the optimal nutritional support among ICUs caring for CHD infants may contribute to poor growth in the period pending surgery [20]. This is of concern, as a poor nutritional status and a low weight at the time of surgery may lead to increased mortality rates [21,22].

Growing literature supports a beneficial role of preoperative feeding [23,24,25,26], which appears to improve postoperative outcomes: CHD infants who were fed before surgery showed more stable postoperative hemodynamics, a better feeding tolerance and wound healing, a decreased duration of mechanical ventilation, a shorter time to reach full calories to wean off parental nutrition, and a shorter hospital stay [19,23,27,28].

Specific feeding protocols have been developed by several institutions caring for CHD patients [10,19,23,27,29,30].

In hemodynamically stable term neonates with or without pharmacological cardiovascular support, the European Society of Paediatric and Neonatal Intensive Care (ESPNIC) recommends to start enteral nutrition within 24 h from admission [31]. Potential contraindications to early enteral feeding are GI anatomic abnormalities, maxillofacial abnormalities, increasing abdominal girth, excessive vomiting and/or diarrhea, positive fecal occult blood test, signs and symptoms of necrotizing enterocolitis (NEC), and lactic acidosis [4,26,32,33,34].

Preoperative enteral feeding should be provided as minimal enteral feeding, with a 10–20 mL/kg/day milk intake for few days [35], aiming to increase enteral intakes by 20 to 30 mL/kg daily until reaching the volume goal [10]. Trophic feeding has been associated with a faster achievement of full enteral and oral feed, more stable hemodynamics, and a shorter need for respiratory support [23]. Although specific data on the benefits of a human milk (HM) diet in infants with CHD are scarce, HM appears to be the preferred option for the initiation of enteral feeds. Given the vulnerability of the GI tract in infants with CHD, it is reasonable that HM could improve GI function and immune maturation, feeding tolerance, and microbiota composition—similar to preterm infants. The optimal caloric intake needed to avoid growth retardation, however, has not been well established [36]. Swartze et al. reported that infants with CHD need about 150 Kcal/kg/day in order to achieve a significant gain of weight, length, and subcutaneous tissue mass [37]. Instead, recent recommendations for moderately malnourished children suggest a caloric intake of 90–110 kcal/kg/day [38], ensuring an enteral protein intake of at least 1.5 g/kg/d to prevent negative protein balance [31].

Marino et al. developed a consensus-based preoperative nutritional strategy for infants with CHD, offering different energy and nutrient intakes (90 to 150 kcal/kg/day and 1.5 to 4 g/kg/day of proteins) according to the infants’ growth pattern; this approach prevented a significant fall in weight-for-ages *z*-scores at 4 months of age and improved weight-for-age and height-for-age *z*-scores at 12 months of age in the intervention group compared to controls [28].

Several concerns exist about possible complications of feeding infants with complex cardiac defects, such as hypoplastic left heart syndrome (HLHS) or single ventricle (SV) [33]. However, preoperative feeding seems to be feasible also in these situations, and some authors have developed specific feeding protocols for the preoperative phase [27]. Among these, Slicker et al. have proposed a nutritional algorithm for infants with HLHS or SV before cardiac surgery, recommending the introduction of enteral feeds in hemodynamically stable patients, even if an umbilical artery catheter (UAC) is in place and prostaglandin (PGE) infusion is ongoing [19]. Furthermore, Fullong-Dillard et al. have found that newborns undergoing biventricular cardiac surgery who received enteral nutrition preoperatively achieved the target feeding volume (135 mL/kg/day) earlier and required postoperative parental nutrition for shorter periods [27].

Food intolerance, fluid intake restriction, and the frequent periods of feeding withholding that characterize the preoperative phase contribute to hinder the achievement of an adequate nutritional support. Disruptions of the enteral feeding plans are frequent among CHD infants: a retrospective study documented that 1 in 5 experienced feeding interruptions, which were mainly related to common procedures such as heart catheterization, brain magnetic resonance imaging, positioning of central lines, gastrostomy and chest tubes, or to the development of GI complications [36]. In a study performed at the Boston Children′s Hospital, the implementation of a nutritional protocol specifically designed to guide clinicians in the feeding management of CHD infants resulted in a significant reduction of feeding interruptions (3 vs. 51 before the feeding protocol introduction, *p* < 0.0001), and in a decreased time to reach the energy targets (1 vs. 4 days, *p* < 0.001), which were achieved by almost all (99%) the infants managed with this new feeding protocol compared to only 31% prior to the protocol introduction [39].

The onset of food intolerance plays a key role in hindering the achievement of full enteral feeding in CHD infants. Signs and symptoms of food intolerance in neonates with heart disease are not well defined [40], and different studies provide different definitions [25,30,39,41,42]. The most common clinical parameters used to identify feeding intolerance are abdominal pain and distension, vomiting, decreased or abolished bowel sounds or movements, and increased residual gastric volumes, which appear as a poor marker to guide the decision to continue, hold, or stop enteral nutrition [18,31,43]. Serum lactate could be used as a marker of intestinal splanchnic perfusion, with raised levels indicating an increased risk of GI complications; however, lactate’s cut-off value predictive of such complications in CHD infants is unknown, limiting the application of this biomarker in routine clinical practice [31]. Near-infrared spectroscopy (NIRS) has been largely adopted in neonatal ICU settings for the continuous monitoring of oxygen delivery at multiple sites, including the splanchnic district [44]; in preterm neonates, splanchnic NIRS monitoring has yield promising results for the prediction of GI complications [45,46,47]. In CHD infants, NIRS-derived mesenteric oxygenation following cardiac surgery has shown a reliable correlation with serum lactate and systemic mixed venous saturation [48]. The role of NIRS for the evaluation of mesenteric oxygenation in relation to enteral feeding in CHD infants has also been investigated. In neonates undergoing biventricular repair or SV palliation, postsurgical NEC development was associated with a significantly lower mesenteric oxygenation at enteral feeding initiation [49]; of note, all the NEC cases occurred in SV infants. Similar results have been also reported by Iliopoulos et al., who monitored mesenteric oxygenation at ICU admission after cardiac surgery on 50 CHD children <10 kg, observing a significant inverse correlation between mesenteric oxygenation at admission and the time needed to establish enteral feeds. Moreover, the authors suggested a cut-off value of 72% be proposed to identify with a 78% sensitivity and 68% specificity infants at risk of developing GI complications—namely, NEC and feeding intolerance [50].

Nutritional status should be assessed by performing serial anthropometric measurements and laboratory investigations aimed at optimizing the nutritional support during hospital stay. Baseline weight, length, weight/length, mid-upper arm circumference, and head circumference, preferably expressed as *z*-scores [18,31], should be recorded at the admission and weekly [10,23]. In CHD infants, total body weight may not reflect the real growth, as they could experience a fluid overload primarily related to the heart condition. The assessment of the body composition could better correlate with clinical outcomes. Body fat mass can be estimated by mid-upper arm circumference, lean mass by body length, and cerebral growth by head circumference [51]; a lower adiposity has been associated with a delayed recovery, a prolonged need for mechanical ventilation, and for vasoactive treatments [52].

Different biochemical parameters should be evaluated to obtain a complete overview of the nutritional status in CHD infants. Serum proteins such as albumin and prealbumin may respectively reflect a chronic or acute undernourishment, which is in turn associated to worse cardiac function [52]. Due to its long half-life (14–20 days), albumin is a marker of chronic malnutrition, although its level can be affected by dehydration, sepsis, trauma, liver disease, and albumin replacement. Prealbumin, also known as transthyretin or thyroxine-binding prealbumin, has a half-life life of 24–48 h and thus reflects acute protein intakes. This protein is synthetized by the liver and excreted by the kidneys, so its values may be altered in cases of renal or liver disease [51,53]. Blood urea nitrogen (BUN) may better correlate with protein intake during enteral feeding in clinically stable newborns; however, BUN may be influenced by renal function, hydration status, and amino acid oxidation for energy production in critically ill patients [54].

Several factors are usually considered when planning to start preoperative enteral feeding. These include, but are not limited to, the risk of NEC, ongoing PGE, and the presence of an UAC.

### 3.1. Necrotizing Enterocolitis

NEC is a rare condition in term newborns without CHD; however, the disease is at least 10–100 times more common in CHD infants [33], with an estimated incidence ranging from 1.6% to 9% [24,33,55,56,57] and mortality rates between 6.8% and 24% [24,55].

The pathophysiology of NEC in term newborns with CHD is different compared to preterm infants: the decreased cardiac output, congestive heart failure, shock, and cardiac surgery and bypass contribute to reduce intestinal perfusion and oxygen delivery, thus making NEC a distinct pathological entity in this population [33,55,58]. Moreover, NEC onset in CHD infants usually occurs in the first seven days of life and typically involves the colon, while in preterm infants the small intestine and ileocecal region are more frequently involved [33,59].

The high risk of NEC in infants with CHD has always represented a limitation to achieve a shared consensus on enteral feeding practice for infants undergoing CHD surgery. However, increasing evidence supports the feasibility of preoperative enteral feeding in neonates with CHD even when the risk of NEC is taken into account [19,23,24,60,61,62], and the use of standardized feeding protocols has proven to reduce the incidence and severity of NEC in this population [63]. Of note, a relationship between time of enteral feeding initiation, increasing feeding volume and velocity rates, the density of milk, and the development of postoperative NEC has not been yet documented [55].

A retrospective study reported a similar prevalence of NEC (approximately 9%) in infants with CHD who received preoperative enteral feeds, either trophic or at higher intakes [24]. Natarajan et al. found only two NEC cases (equal to 3% of the studied population) in their cohort of CHD infants who received an intake of at least 100 mL/kg/day before surgery [61].

The risk of NEC is higher in infants with ductal-dependent anomalies, such as critical aortic stenosis, interrupted aortic arch, coarctation of the aorta, and HLHS; in particular, the latter condition has been associated with an estimated NEC incidence ranging between 7.6% and 18% [32,33,56,64]. The decision about feeding these infants before surgery is usually based on the experience of the center and of clinicians, and also relies on the assessment of the characteristics of the transductal shunt and on the abovementioned biochemical parameters [65]. In a large Swedish cohort of 444 newborns with ductal-dependent systemic circulation, 46 out of 47 infants with HLHS were enterally fed prior to surgery, receiving at least 45 mL/kg/day of milk, and no cases of preoperative NEC were observed [60].

Given the different etiology, both short-term complications, such as bowel perforation, need for surgery, and sepsis, and long-term issues (e.g., development of strictures or short bowel syndrome) are less common in CHD infants compared to those without this condition [66]. Furthermore, the occurrence of NEC in infants with CHD has not been associated with increased mortality rates, regardless of CHD type and severity [56,57]. A potential explanation for the better prognosis of NEC in this population could be related to the close clinical monitoring of these infants, which allows a prompt NEC diagnosis and intervention [57,66].

### 3.2. Treatment with Prostaglandin

PGEs are a group of physiologically active lipid compounds called eicosanoids that are used to maintain the ductus arteriosus patent in neonates with ductal-dependent CHD in order to promote mixing of pulmonary and systemic blood flow or to improve pulmonary or systemic circulation [67]. Nevertheless, the resulting hemodynamic disturbances of post-ductal systemic blood flow may lead to splanchnic hypoperfusion, thus increasing the risk of NEC [68]. Hence, due to the potential risk of intestinal complications, many clinicians prefer to avoid enteral feeding during PGE infusion [19,36,55,65].

Willis et al. have reported adequate feeding tolerance in term infants with duct-dependent CHD during PGE treatment, except for one infant with bidirectional shunt through the ductus arteriosus [25]. As to NEC, Becker et al. found a 0.3% incidence of this condition in term infants with ductal-dependent CHD who were enterally fed while on PGE [69], and Day et al. observed an even lower incidence in their cohort, documenting no association between enteral feeding and NEC occurrence during PGE treatment [70].

### 3.3. Presence of an Umbilical Arterial Catheter

A UAC in place represents another postulated risk factor for the development of NEC: by reducing the aortic lumen, the UAC may decrease the superior mesenteric artery blood flow, thus increasing the risk of mesenteric arterial thrombosis [71,72,73]. In some ICUs, enteral feeding is avoided as long as the UAC is in place [30,65], whereas in other settings, feeding in the presence of UAC is considered a safe practice. One trial [74] and a prospective observational study [75] in preterm neonates found that having a UAC in situ did not affect mesenteric blood flow or lead to greater rates of feed intolerance or NEC. In the study by Alten et al., 76 out of 99 term CHD infants with a UAC in place received enteral feeds pre- and/or postoperatively: of the 8 cases of NEC documented in the whole study cohort, consisting of 251 infants, only 2 occurred in the UAC cohort [16].

The very recent position statement by the ESPNIC strongly supports the provision of enteral nutrition in term neonates with CHD, even with a UAC in place and during PGE infusion, providing a close monitoring of the infants [31].

## 4. Postoperative Feeding Management

Due to the high incidence of growth failure and GI complications in infants requiring surgery for CHD, there has been great interest in developing specific postoperative feeding protocols. Braudis et al. reported that the implementation of a postoperative feeding protocol in infants with HLHS decreased the incidence of late onset sepsis, the time to achieve the goal caloric intake, and the need for parental nutrition when compared to historical controls [26].

In the postoperative period, it is common for CHD infants to have an open sternotomy for several days and to experience extracardiac complications such as respiratory failure, chylothorax, renal failure, and neurologic impairment, thus raising concerns for enteral feeding. As per preoperative feeding, an early initiation of feeding after cardiac surgery and the existence of a postoperative feeding protocol have been associated with improved growth [26] and decreased length of hospital stay [30]. However, since there are few data evaluating the relation between postoperative feeding and outcomes, practices on feeding initiation and advancement after CHD surgery are often anecdotal and institution-based [10]. Consistently, Alten et al. observed a great variability in feeding practices across cohorts and centers. The timing for starting postoperative enteral feeding varied significantly, with a median time of 2 (range 1–4) postoperative days. Initiation of postoperative feeds was delayed in patients after stage I palliation for HLHS, who are often hemodynamically unstable and require vasoactive medications and mechanical ventilation for prolonged periods. Postoperative feeds were initiated in 35% of patients before extubation (range across institutions: 21–61%). Patients who underwent aortopulmonary shunt placement received enteral feeding 19% of the time during their ICU stay, and 55% of patients with HLHS were fed while mechanically ventilated [16].

Schwalbe-Terilli et al. evaluated the caloric intake of 100 neonates receiving enteral nutrition after cardiac surgery. The patient group were separated into biventricular cardiac defects and functional single ventricle. The center practice was to avoid enteral feeds in infants on PGE or with a UAC. Their practice was to advance feeds slowly over a period of 48 to 72 h to a volume of 100 mL/kg/d, then increasing caloric density to 24 to 27 kcal/oz with a goal of 120 to 150 mL/kg/d. Feeds were initiated as boluses given through a nasogastric tube before attempting oral feeding. In this study, a caloric intake of 100 kcal/kg was achieved on 48.4% of days of enteral feeding, and 120 kcal/kg was achieved in only 19.7% of feeding days. The authors reported a median weight change during the period of enteral feeding of −20 g, which was likely affected by their practice of discontinuing parenteral nutrition (PN) at 100 mL/kg/d of enteral nutrition or when the central venous line was removed [36].

The exact caloric requirements of infants with CHD in the postoperative period are difficult to establish. Commonly used predictive equations do not provide accurate estimates of energy requirement in individual patients during the highly dynamic postoperative phase. Although indirect calorimetry can best estimate energy requirements in children with CHD, it is available in a minority of ICUs, and its applicability during the acute postoperative phase is hindered by high FiO2 requirements, endotracheal tube leaks, pleural air leaks, or intracardiac shunting [31]. Measurement of nitrogen balance is the recommended method to determine minimal protein requirement. During the early postoperative period, daily measurements of resting energy expenditure using indirect calorimetry and of nitrogen balance in each individual child would be of great value to optimize energy and protein intakes in order to meet their requirements [76].

With regard to the role of type of cardiac defect on enteral feeding management, a few previous studies have examined the incidence and clinical impact of GI morbidity among neonates with HLHS following the first stage palliation [32,77]. In the study by Jeffries and colleagues, GI complications occurred in 48/117 infants and included NEC, need for home feeding tubes, and prolonged hospital stay for nutritional support [32]. In another study comparing infants with HLHS and transposition of great arteries (TGA), the time needed to achieve full caloric intakes was significantly longer in the HLHS group (24 vs. 12 days, respectively; *p* < 0.001) [77]. In addition, infants with HLHS had a higher incidence of feeding-related complications such as reflux disease, aspiration, and poor suck/swallow compared to those with TGA (48% vs. 4%, respectively; *p* = 0.001). A significantly higher proportion of the TGA infants (76.9% vs. 25.9%) were fed orally at discharge [32,77].

## 5. Breastfeeding and Role of Milk Type

The exclusive source of nourishment recommended by the World Health Organization up to 6 months of life and as a complementary food for the first two years of life is breast milk [78], thanks to its nutritional and functional benefits [79]. Despite evidence supporting the beneficial effects of HM in vulnerable high-risk population such as preterm infants, there have been few studies assessing the benefits of HM in infants with CHD. A single-center retrospective cohort study of 546 infants with complex CHD evaluated potential feed-related risk factors for NEC in the preoperative period. The authors found that an exclusive unfortified HM diet was associated with a significantly lower risk of preoperative NEC in a multivariable regression model controlling for cardiac lesion, race, feeding volume, birth weight, gestational age, presurgery, and pre-NEC inotrope use [80].

Nevertheless, the rate of direct breastfeeding in infants with CHD is low due to the risk of aspirations due to pharyngo-laryngeal incoordination and the concerns related to their hemodynamic instability [81]. Despite these concerns, a comparative study between breast-fed and formula bottle-fed infants reported that not only children with CHD were able to breastfeed, but they also had a better weight gain compared to bottle-fed infants [82]. Since many of these newborns need a restriction of fluids and receive diuretic therapy, a precise monitoring of the intake volume is mandatory. The exact quantification of enteral volumes can be achieved also in breastfed infants: according to some authors, the breastfeeding weight test, consisting of the weight difference before and after feeding, could be useful in this respect and would also encourage the mother to provide her own milk [81].

Despite HM benefits, mother’s own milk (MOM) provision to critical infants with CHD is hindered by several factors, such as the separation of the mother–infant dyad after delivery, the stressful postpartum environment for both the mother and the infant, and the lack of lactation support. Maternal support, prenatal breastfeeding education, mothers’ access to a hospital-grade breast pump and facilities, and medical staff education have all been found to be effective in promoting HM provision to infants with CHD [83].

When MOM is not available soon after birth or is not sufficient to fulfil enteral requirements, a reasonable alternative could be donor milk (DHM). In analogy with preterm infants, for whom DHM constitutes the optimal alternative to MOM, the use of DHM should also be implemented for CHD infants in light of its role in promoting feeding tolerance and reducing the risk of NEC compared to formula [84,85]. In a very recent single-center, retrospective cohort study, the impact of a multi-interventional nutrition program on clinical outcomes in newborns facing surgery for CHD was evaluated: after the introduction of a DHM program, significantly more patients received DHM or expressed breast milk in both the preoperative and postoperative periods. Moreover, weight-for-age *z*-score improvement from birth to hospital discharge obtained with the institution of a postoperative feeding protocol were maintained after the introduction of the DHM program [62].

Since infants with CHD have a high energy expenditure and need fluid intake restriction as part of their management to prevent adverse clinical outcomes, including NEC and mortality, several studies have evaluated the potential role of high-calorie formulas to reach the nutritional requirement needed for proper growth. The use of an energy enriched formula or a protein dense formula, compared to standard formula (1.4 g/100 mL, 67 kcal/100 mL), resulted in a faster achievement of the nutritional goal with a higher weight gain and increased levels of serum albumin and amino acids. The only documented side effect was an initial diarrhea, probably related to the higher osmolarity of the enriched formula [42,86,87]. On the contrary, an observational study including 122 infants with biventricular and univentricular cardiac defects undergoing surgery showed no difference in postoperative growth and hospitalization length between infants receiving MOM, a standard formula (67 kcal/100 mL), or a preterm formula (80 kcal/100 mL) [88].

## 6. Feeding Issues

Feeding issues and delays in achieving full oral nutrition are frequent in infants with CHD and are still observed at two years of age in 22% of infants undergoing neonatal cardiac surgery [89]. Thus, many children depend on prolonged enteral nutrition, either through the nasogastric tube or the gastrostomy, to meet their nutritional needs during hospitalization and often also after hospital discharge [8,9,16].

The type and severity of heart disease affects the acquisition of feeding skills; specifically, children with cyanotic heart lesions face longer delays in taking their first oral feed and in reaching the goal intake volume compared to children with non-cyanotic heart disease [90]. Children with single ventricle physiology, especially HLHS, often need prolonged enteral feeding, either through a nasogastric tube or a gastrostomy [7,61,77,91,92]. A more complex surgical approach even with the use of cardiopulmonary bypass turns out to be decisive in increasing the time to achieve full oral feeds [9,90]. Comorbidities such as gastroesophageal reflux disease, postoperative vocal cords paralysis, genetic syndromes, and a longer need of interrupting enteral feeds are associated with the need for device-assisted feeding and are predictors of reduced oral feeding capacity [91,93]. Hospital-dependent factors also influence the development of oral feeding skills, including the implementation of preoperative feeding [93] and the time to initiate postoperative enteral nutrition [91,94]. Instead, there are controversies in the literature about the role of prolonged intubation duration, as most studies describe invasive respiratory support as a risk factor for developing feed difficulties [9,90,91,94,95], while a study claims it has no influence on oral motor skills [96].

It is unclear whether there is an optimal route in terms of efficacy and safety for providing enteral feeding to CHD infants, and this leads to a high variability between centers in choosing a nasogastric tube or a gastrostomy [16]. In a cohort of infants with single ventricle heart defects, no significant association was found between feeding modality and change in weight-for-age *z*-score after stage 1 surgery [97]; other authors found preemptive gastrostomy placement to be associated with improved survival to stage 2 surgery operation but not with shorter hospitalization or better growth [98].

The assessment of oro-motor skills, aspiration risks and the consequent application of a specific intervention to improve oral feeding skills in newborns facing surgery for CHD has resulted in improved weight gain and fewer gastrostomy placements at discharge [62,91].

Children with CHD have an increased risk of abnormal neurological development since arterial saturation and brain oxygenation associated with neurological development can be compromised by unstable hemodynamics [99]. Growing literature has highlighted the impact of feeding mode on neurodevelopmental outcomes in children with CHD [100]. The inability to achieve full oral nutrition and the prolonged need for enteral feeding tubes or gastrostomy are associated with delays in cognitive, communicative, and motor domains at 6, 12, and 24 months of age [8,101,102,103].

Nutritional issues in infants with CHD may persist after hospital discharge. Several studies documented a high rate of failure to thrive in infants with CHD after discharge, both in severe and milder forms [8].

Hence, during hospital stay and after discharge it is necessary to perform a close monitoring of growth and nutrition of infants with CHD, especially in the first year of life, since longitudinal and head circumference growth may experience delay for one year or more [85].

## 7. Conclusions

Despite advances in neonatal and cardiac intensive care, infants with CHD have a greater risk of malnutrition and growth deficit, as well as of negative long-term neurodevelopmental outcomes. Although optimal nutrition is considered essential to improve outcomes in critically ill infants, there is lack of high-quality evidence to guide enteral nutrition in this high-risk population with increased nutritional challenge. Therefore, a wide variability in feeding management exists worldwide, across clinicians, institutions, and countries. Pathophysiology of nutritional and metabolic changes is age dependent. However, available observations and recommendations for cardiac infants partially rely on studies in preterm infants, as evidence from term infants is scarce. Acknowledging these limitations, currently available literature highlights the role of nutritional management and suggests changes in current practice that are likely to improve nutrition status and outcomes of infants with CHD (Box 1). Large, well-designed studies assessing standardized clinically relevant outcomes are needed to provide robust evidence and shared recommendations and to achieve an implementation of evidence into current practice.

Box 1 **Suggested approaches emerged from current literature to improve nutritional status and out-comes in infants with congenital heart disease.**

- Implementation of enteral feeding protocols, both preoperative and postoperative, with clearly stated reasons to withhold enteral feeds and definitions of feeding intol-erance.
- Early minimal enteral feeding within 24 h of admission.
- Enteral feeding should be considered in term infants with umbilical arterial catheters and during prostaglandin infusion.
- Enteral feeding should be considered in stable term infants on hemodynamic support.
- Frequent assessment of nutritional status and growth parameters.
- Assessment and support of oro-motor skills.
- Promotion and support of breastfeeding and provision of mother’s own milk (MOM); if MOM is not available, consider donor milk (if available).
